# Neighborhood social capital is associated with participation in health checks of a general population: a multilevel analysis of a population-based lifestyle intervention- the Inter99 study

**DOI:** 10.1186/s12889-015-2042-5

**Published:** 2015-07-22

**Authors:** Anne Mette Bender, Ichiro Kawachi, Torben Jørgensen, Charlotta Pisinger

**Affiliations:** Research Centre for Prevention and Health, Building 84/85, Glostrup Hospital, DK-2600 Glostrup, Denmark; Harvard School of Public Health. Department of Social and Behavioral Sciences, 677 Huntington Ave., 7th floor, Boston, MA 02115 USA; Faculty of Health Science, University of Copenhagen, Copenhagen, Denmark; Faculty of Medicine, University of Aalborg, Aalborg, Denmark

## Abstract

**Background:**

Participation in population-based preventive health check has declined over the past decades. More research is needed to determine factors enhancing participation. The objective of this study was to examine the association between two measures of neighborhood level social capital on participation in the health check phase of a population-based lifestyle intervention.

**Methods:**

The study population comprised 12,568 residents of 73 Danish neighborhoods in the intervention group of a large population-based lifestyle intervention study - the Inter99. Two measures of social capital were applied; *informal socializing* and *voting turnout*.

**Results:**

In a multilevel analysis only adjusting for age and sex, a higher level of neighborhood social capital was associated with higher probability of participating in the health check. Inclusion of both individual socioeconomic position and neighborhood deprivation in the model attenuated the coefficients for *informal socializing*, while *voting turnout* became non-significant.

**Conclusion:**

Higher level of neighborhood social capital was associated with higher probability of participating in the health check phase of a population-based lifestyle intervention. Most of the association between neighborhood social capital and participation in preventive health checks can be explained by differences in individual socioeconomic position and level of neighborhood deprivation. Nonetheless, there seems to be some residual association between social capital and health check participation, suggesting that activating social relations in the community may be an avenue for boosting participation rates in population-based health checks.

**Trial registration:**

ClinicalTrials.gov (registration no. NCT00289237).

**Electronic supplementary material:**

The online version of this article (doi:10.1186/s12889-015-2042-5) contains supplementary material, which is available to authorized users.

## Background

Participation in population-based preventive health check have declined over the past decades [5]. Low participation may partly explain why population-based general health check have been ineffective in preventing disease at the population level [12, 18]. Non-participants typically come from lower socioeconomic backgrounds and have higher risk of undetected diseases [1, 5]. The higher participation of people in health check programs who are already at low risk is an illustration of the inverse care law [9]. Furthermore, new research has begun to highlight lower participation among persons living in deprived neighborhoods, which goes beyond the effect of individual socioeconomic position [2, 24, 29].

Social capital as a by-product of social relationships arising from reciprocal exchange between members engaging in social interactions within a neighborhood or network [14] has been studied extensively in relation to public health [24, 27]. The “capital” in social capital refers to various resources that are accessed through social networks, including health-relevant information, instrumental resources, and affective support [20]. Social capital can be conceptualized at both the individual and collective levels. An individual who has “high social capital” is someone who is endowed with an abundance of social connections that can provide access to resources. At the collective level, a community that is endowed with high stocks of social capital is one in which relations between residents are characterized by high levels of trust, mutual assistance, reciprocity, and collective ability to undertake action for the common good [13].

There is considerable heterogeneity in the indicators used to measure neighborhood social capital, which is largely a product of the reliance of investigators on proxy indicators derived from secondary sources of data (i.e. surveys carried out for purposes other than public health) [16]. While cognitive social capital refer to norms, attitudes, values and beliefs, structural components refer to objective aspects of social organization, such as the density of social networks and patterns of civic engagement [8]. In this paper we focused on two structural indicators of social capital; *informal socializing* and *voting turnout. Informal socializing* within neighborhoods is the primary mechanism through which residents exchange resources, for example, instrumental support, health-relevant information, and affective support [20]. Within social capital research, groups and network membership is the dimension most commonly included in indexes of social capital [7]. Typically, this dimension is assessed by means of questions covering the nature and extent of a person’s participation in various types of social organizations and informal networks. Informal network access commonly include—ties to people who are similar in terms of their demographic characteristics, such as family members, neighbors, close friends and work colleagues [25]. Informal social networks from a web of associations provide support and serve to shape norms of behavior, and they have the capacity to produce social capital [7]. To the extent that social capital is considered stocks of social capital accumulated among residents of a neighborhood; social capital obtained within one network (e.g. tight family network) therefore may be transferred to other domains (e.g. neighbors and local community).

The second measure used in this paper is the extent of political participation of the neighborhood, measured by *voting turnout*. This measure is increasingly being applied as a proxy measure for social capital [10, 22, 25, 28]. The empirical research of Sidney Verba and colleagues has demonstrated a tight connection between social capital and political activity. This may happen through at least three distinct mechanisms: a) citizens become psychologically engaged in politics through their informal social contacts; b) social networks serve as a locus for recruitment into political activities (e.g. organizing protests and petitions, or encouraging friends to vote); and c) by participating in various social groups (e.g. church groups, hobby groups, neighborhood associations), citizens acquire organizational skills that are directly transferable to politics, even though the civic groups may not have any political agenda [28].

To our knowledge, no previous study has examined the relation between community social capital and participation in general health checks. One cross-sectional study on participation in cancer screening showed higher participation rates in women with high perceived social capital [2]. Social capital is suggested to partly mediate the effects of socioeconomic position on health-related behaviors [6, 21, 23] and mortality [15] and we therefore hypothesized that a high level of social capital, measured as either *informal socializing* or *voting turnout*, increases participation in general health checks.

Accordingly the primary objective of this study was to test the effect of two measures of neighborhood level social capital (*informal socializing* and *voting turnout*) on participation in the health check phase of a population-based lifestyle intervention.

## Methods

### The Inter99 study

Inter99 study was a population-based randomized lifestyle intervention with a catchment area covering 73 neighborhoods (census districts) in the south-western part of Copenhagen County, Denmark. Mean adult neighborhood population size was 2457 persons (range: 464–5412). The adult working-age population (25–65 years) of this area included 179,359 persons. The design of the study has previously been described in detail [11]. The study population consisted of a stratified sample of all inhabitants born in 1939–40, 1944–45, 1949–50, 1954–55, 1959–60, 1964–65, and 1969–70 (n = 61,301) and these were pre-randomized (December 2nd 1998) to either control (n = 48,285) or intervention (n = 13,016) group. At baseline all persons in the intervention group were invited to health checks and assessment of 10-year risk of ischemic heart disease at the Research Centre for Prevention and Health taking place between March 15th 1999 and January 31st 2001. They all had lifestyle counseling of varying intensity according to their assessed risk [11] and persons at high risk of IHD were additionally over a four to six month period offered six sessions of group-based counselling. All persons in the intervention group received questionnaires regarding health and lifestyle.

The analyses of this paper are based on all persons in the intervention group. A total of 88 persons in the intervention group emigrated, were lost to follow-up or died in the period between date of randomization and baseline. Furthermore, between date of randomization and January 1^st^ 1999, when data on census district was retrieved from the registers a total of 76 persons moved to a municipality outside the study area and we were not able to identify the census district of 84 persons (1 %). Additionally, educational attainment was missing for 204 persons (2 %), leaving 12,564 persons for analyses.

All participants gave a written consent before taking part in the study. In Denmark researchers have permission to use registers for research purposes without persons’ informed consent as long as they comply with predefined research regulations, which made it possible to obtain register information on participants as well as non-participants. The study was approved by the Regional Scientific Ethics Committee (KA 98 155) and the Danish Data Protection Agency and is registered at ClinicalTrials.gov (registration no. NCT00289237).

### Individual level factors

*Participation:* Persons were categorized as participating if they attended the health check.

*Educational attainment* was categorized into *basic* education (up to high school), *low* education (<2 years of vocational training), *middle* education (2–4 years of vocational training/education), and *high* education (>4 years; academic degree).

*Income* (equalized disposable income) was calculated as the average household income after taxation and interest, divided by the number of equivalent adults in the household. Equalized family size was calculated as follows; the first adult was given a weight of 1.0, each subsequent adult was given a weight of 0.5 and each child under 14 years was given a weight of 0.3 [4]. Furthermore, income was corrected for inflation by adjusting to the year 2000 price index. As income was not normally distributed, the variable was divided into quartiles.

*Employment status* was categorized into *wage earners*, and persons *out of workforce* (e.g. students, retired, unemployed).

*Covariates:* Data on personal identification number, age and sex was retrieved from the Central Personal Registry. In Denmark each person is assigned a unique identification number at birth which enables citizens to be followed for the rest of their life.

### Neighborhood level factors

*Neighborhood informal socializing:* A total of 6537 persons responded to two items regarding their social networks;

- *“How often do you have contact with family members, with whom you do not live?”*
- *“How often do you have contact with friends or acquaintances?”*

For both questions, the five response categories were given scores as follows: daily (4), several times a week (3), several times a month (2), hardly ever (1) and never (0). All respondents were assigned to their respective neighborhoods and the mean contact frequency with family and friends respectively was calculated at the neighborhood level. On average 91 persons (range: 18–240 persons) from each neighborhood completed the two questions. The 73 neighborhoods were ranked according to their mean friend contact frequency and were divided into quartiles. Corresponding procedure was conducted for family contacts. In order to construct a variable for *informal socializing* we summed the quartile-scores of the two variables which resolved in a score with values ranking from 2 to 8 (8 being high socializing). This score was divided in four levels of informal socializing; *very low* (scores 2 + 3, n = 19 neighborhoods), *low* (score 4 = 15 neighborhoods), *middle* (scores 5 + 6, n = 22 neighborhoods) and *high* (scores 7 + 8, n = 17 neighborhoods).

*Neighborhood voting turnout:* Voting turnout for each of the 73 neighborhoods was used as the second measure of social capital. Voting turnout (in %) in the elections for the Danish parliament on November 20^th^ 2001 was used for this purpose [17] and was based on all persons residing in the neighborhoods. In the multilevel analyses, all of the 73 neighborhoods were ranked and divided into quartiles according to their level of voting participation (*very low* (<83 %)*, low* (83–85 %)*, middle* (86–89 %)*, high* (>89 %)).

*Neighborhood deprivation:* The income of all persons between the ages of 25 and 65 who by January 1^st^ 1999 were living in the Inter99 study area (n = 179,097) was ranked and divided into quartiles. All persons were grouped into their respective census districts (n = 73) and the districts were ranked according to the proportion of persons with an income within the lowest quartile (family disposable income <16,500$/year). We then divided the districts into quartiles, creating a neighborhood deprivation variable with four levels of deprivation; *very low, low, middle* and *high*.

### Statistical analyses

Data from registers was merged with data from the Inter99 study by using census districts and individual identification numbers as key variables. Descriptive statistics include mean neighborhood participation in relation both to *voting turnout* and *informal socializing* (defined as proportion never/hardly ever seeing friends or family). Also, contingency tables were conducted showing distribution of baseline characteristics (sex, age, educational attainment, employment status, income, neighborhood deprivation) in neighborhoods with very low and high *informal socializing* and likewise in neighborhoods with very low and high *voting turnout.* Additionally, a *p*-value for chi-square was calculated for each covariate, indicating if the distribution of persons within the categories of each covariate varied significantly between neighborhoods with different level of social capital. As there were only marginal differences in the baseline analyses between men and women, all analyses were conducted for men and women combined.

We estimated the relative risks (RRs) of health check participation according to the level of *informal socializing* and *voting turnout* by conducting multilevel analyses with binomial distributions and log links. Census district code was included in the random statement to account for intra-neighborhood correlation and the intraclass correlation coefficient (ICC) was calculated as: $\frac{\sigma^2}{\sigma^2+1}$. *P*-value for difference between each category and the reference category was calculated together with 95 % confidence intervals and a *p*-value for a trend between level of social capital and participation. Model 0 included sex and age in addition to the two measures of social capital. In model 1 three measures of individual socioeconomic position (education, income and employment status) were added as they could confound the effect of social capital on participation. Model 2 included only neighborhood deprivation, age and sex as confounders and model 3 included all three measures of socioeconomic position, neighborhood deprivation, age and sex.

All analyses were performed through the use of the statistical software SAS (version 9.3; SAS Institute).

## Results

**Fig. 1:**
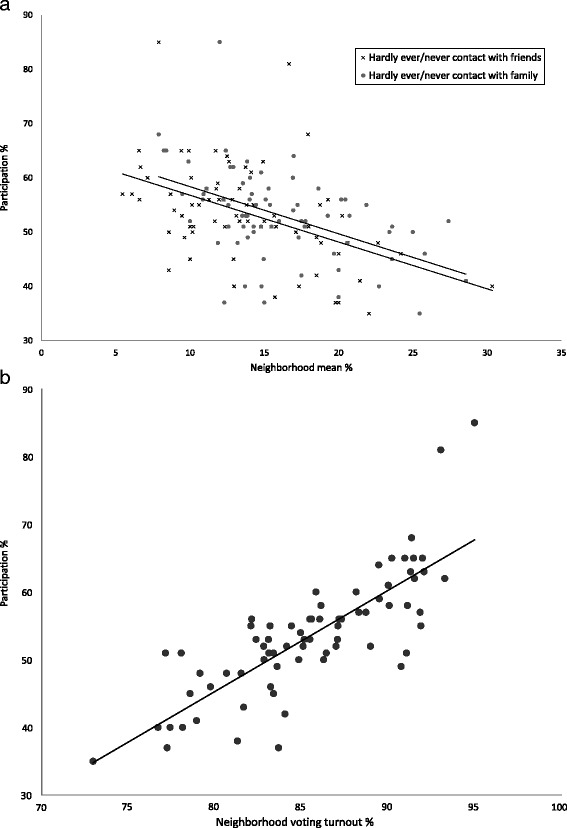
**a**. Neighborhood informal socializing: Mean neighborhood (n = 73) study participation according to neighborhood percentage who hardly ever/never have contact to friends and family. **b**. Neighborhood voting turnout: Mean neighborhood (n = 73) study participation according to neighborhood voting turnout

The mean health check participation rate ranged from 35 to 85 % between neighborhoods (population mean = 52.5 %). A decrease in the level of the two measures of informal socializing was associated with a steady decrease in mean health check participation (Fig. 1a). Likewise, there was a clear linear dose response relationship between voting turnout and mean health check participation (Fig. 1b).

Persons living in neighborhoods with very low *informal socializing* and very low *voting turnout* had when compared to those living in neighborhoods with high *informal socializing* and high *voting turnout* on average lower education, lower income, more were out of workforce and more were living in deprived neighborhoods (Table 1).

**Table 1:** Distribution (in %) of baseline characteristics among persons living in neighborhoods with very low or high *informal socializing* and very low or high *voting turnout*

	Informal socializing		Voting turnout	
	Very low	High	Very low	High
Gender				
Men	50	50	51	50
Women	50	50	49	50
*P*-value	0.921		0.084	
Age				
30 or 35	32	27	33	25
40, 45 or 50	37	38	38	38
55 or 60	31	35	29	37
*P*-value	<0.001		<0.001	
Education				
Basic	36	24	37	23
Low	50	56	49	56
Middle	10	14	10	16
High	4	6	4	5
*P*-value	<0.001		<0.001	
Income				
I-Lowest quartile	31	17	34	16
II	26	23	26	23
III	24	27	23	26
IV-Highest quartile	19	33	17	35
*P*-value	<0.001		<0.001	
Employment status				
Out of workforce	24	11	27	10
Wage earner	76	89	73	90
*P*-value	<0.001		<0.001	
Neighborhood deprivation				
High	36	0	69	0
Middle	49	30	23	0
Low	15	16	3	19
Very low	0	59	0	81
*P*-value	<0.001		<0.001	

Participation in the health check varied on average by 1.2 % (ICC = 0.012, SE = 0.004) between neighborhoods when adjusting for age and sex. In a model including both *informal socializing* and *voting turnout*, and only age and sex as potential confounders (Table 2, Model 0), there was a clear significant increase in probability (relative risk [RR]) of participating with higher level of *informal socializing* and *voting turnout*. Persons living in neighborhoods with the highest level of *informal socializing* had 14 % higher probability of participating and those living in neighborhoods with the highest level of *voting turnout* had 26 % higher probability of participating, when compared to those living in neighborhoods with the lowest level of social capital.

**Table 2:** Probability (RR, CI 95 % *p*-value) of participating by *informal socializing* and *voting turnout* (Model 0) plus individual socioeconomic position (Model1), plus neighborhood deprivation (Model2) and both individual socioeconomic position and neighborhood deprivation (Model 3)

	Model 0				Model 1				Model 2				Model 3			
	RR	CI 95 %		*P*-value	RR	CI 95 %		*P*-value	RR	CI 95 %		*P*-value	RR	CI 95 %		*P*-value
Neighborhood level factors																
Informal socializing																
*High*	1.14	1.08	1.22	<0.001	1.09	1.03	1.16	0.003	1.12	1.05	1.20	<0.001	1.08	1.02	1.15	0.009
*Middle*	1.09	1.02	1.16	0.011	1.05	0.99	1.12	0.104	1.07	1.00	1.14	0.045	1.04	0.98	1.11	0.172
*Low*	1.07	1.02	1.13	0.009	1.05	1.00	1.10	0.061	1.07	1.01	1.13	0.017	1.04	0.99	1.10	0.098
*Very low*	1	(ref.)			1	(ref.)			1	(ref.)			1	(ref.)		
*P*-value for trend	<0.001				0.031				0.005				0.068			
Voting turnout																
*High*	1.26	1.19	1.33	<0.001	1.11	1.05	1.18	<0.001	1.09	0.99	1.20	0.0871	1.06	0.97	1.16	0.212
*Middle*	1.14	1.08	1.20	<0.001	1.05	0.99	1.10	0.080	1.02	0.93	1.11	0.740	1.01	0.93	1.10	0.846
*Low*	1.08	1.03	1.14	0.002	1.01	0.96	1.06	0.678	1.00	0.93	1.07	0.974	1.00	0.93	1.07	0.896
*Very low*	1	(ref.)			1	(ref.)			1	(ref.)			1	(ref.)		
*P*-value for trend	<0.001				<0.001				0.031				0.167			
Neighborhood deprivation																
*Very low*									1.19	1.08	1.31	<0.001	1.06	0.97	1.16	0.204
*Low*									1.14	1.05	1.25	0.002	1.05	0.97	1.14	0.267
*Middle*									1.11	1.03	1.19	0.006	1.01	0.95	1.08	0.747
*High*									1	(ref.)			1	(ref.)		
*P*-value for trend									0.005				0.524			
Individual factors																
Education																
*High*					1.17	1.08	1.26	<0.001					1.17	1.08	1.26	<0.001
*Medium*					1.27	1.20	1.33	<0.001					1.27	1.20	1.33	<0.001
*Low*					1.18	1.13	1.23	<0.001					1.18	1.13	1.23	<0.001
*Basic*					1	(ref.)							1	(ref.)		
*P*-value for trend					<0.001								<0.001			
*Wage earner*					1.31	1.26	1.35	<0.001					1.31	1.26	1.35	<0.001
*Out of workforce*					1	(ref.)							1	(ref.)		
*P*-value for trend					<0.001								<0.001			
Income																
*I-Highest quartile*					1.32	1.25	1.40	<0.001					1.32	1.25	1.39	<0.001
*II*					1.22	1.15	1.29	<0.001					1.22	1.15	1.29	<0.001
*III*					1.16	1.10	1.23	<0.001					1.16	1.10	1.23	<0.001
*IV-lowest quartile*					1	(ref.)							1	(ref.)		
*P*-value for trend					<0.001								<0.001			
ICC (SE)	0.004	(0.002)			NS				0.003	(0.001)			NS			

*RR* relative risks, *NS* not significant, *ICC* intraclass correlation coefficient, *SE* Standard error

After including socioeconomic position in the model (Table 2, Model 1) or neighborhood deprivation (Table 2, Model 2) the effects of *informal socializing* and *voting turnout* on health check participation were considerably attenuated, but nonetheless remained statistically significant. In model 1 and model 3, when including simultaneously for social capital indicators and individual socioeconomic position, the random effect of neighborhood became non-significant due to low intra-neighborhood variability. The random statement was therefore omitted in Table 2, model 1 and model 3. In model 3 (Table 2) after simultaneously including socioeconomic position, neighborhood deprivation, age and sex, the effect of *voting turnout* on health check participation rendered statistically non-significant. However, health check participation rates for neighborhoods with high *informal socializing* remained significantly higher than neighborhoods with very low *informal socializing*, albeit somewhat more weakly associated than in the previous models.

## Discussion

In this paper we found that higher level of neighborhood social capital was associated with higher probability of participating in the health check phase of a population-based lifestyle intervention. Most of this association was explained by individuals residing in deprived neighborhoods and those with lower socioeconomic position also living in neighborhoods with low social capital.

The results of this paper bring new insights to the evidence on the linkage between social capital and health behavior. To our knowledge no other study has examined the effects of social capital on participation in general health checks. Our results are in line with studies demonstrating a link between social capital and other health maintenance behaviors [6, 21, 23], indicating that high social capital promotes positive health behaviors. One explanation of the results of this paper may be that residents of deprived neighborhoods are more disconnected from sources of information, or lack the social reinforcement and social support to attend a health check. In a previously published paper it was shown that neighborhood deprivation predicts participation above and beyond the effects of individual socioeconomic position [2]. However, in this paper we find no significant effects of neighborhood deprivation in the model that included social capital and individual socioeconomic position. This result may be explained by all of the effect of neighborhood deprivation on health check participation being mediated through social capital.

One argument against our interpretation is that voting participation and participation in a health check are tapping into the same thing – that is, both are measuring a tendency toward higher civic engagement. We argue, however, that political participation and participation in a health check are not interchangeable with each other. Our theoretical model is that the quality of neighborhood social relations (i.e. social capital) is “upstream” of both political participation and health check participation. According to this view, community social capital is the force that mobilizes residents to both get out and vote, as well as to participate in health check. Of course, we hasten to add that factors other than social capital also drive health check participation, such as health status, interest in health, family disposition for ischemic heart disease, previous experiences with the health care system and perceived authority of researchers and health care professionals. We did not consider these factors potential confounders as they are unlikely to influence the level social capital.

If our theoretical model is correct, then strengthening neighborhood social bonds may prove to be a viable approach for boosting the rate of participation in community-based health checks and health promotion interventions. If an intervention was able to increase the neighborhood social capital, measured as either *informal socialization* or *voting turnout*, from the lowest to the highest level, we would expect study participation to increase by 9-11 %, after taking into account differences in individual socioeconomic position. We still have limited knowledge on how social capital could be mobilized in local communities. However, a recently published study from Sweden showed that increasing social capital through human interaction is likely to have health promoting effects [3]. The authors suggested a number of interventions which included building neutral meeting places, green areas and walking-friendly neighborhoods.

In this paper, we adopted two measures of neighborhood social capital; *informal socializing* and *voting turnout.* A limitation of the measure of contact frequency with friends/family is that non-respondents did not contribute to the mean. Including information from non-participants would have produced more precise estimates, however the method did not seem to bias the results, as the residuals of the outcome did not vary according to the level of *informal socializing*. Furthermore, if there is a high correlation between participant’s individual social network and that of their neighbors, the measure may in fact not reflect a collective measure of *informal socializing* but, rather than an average of that of the residents. Thus, even though we demonstrate that high stocks of social capital concentrated within a neighborhood are correlated with participation in a health check, we cannot be sure that this is explained by the social capital of a collective nature. Least, though we assume that high stocks of social capital, reflected in a high contact frequency with family, is likely to raise the stocks of social capital of the neighborhood in general, this is not documented in detail in the literature. Additional statistical analyses (Additional file 1) in which the the measure of *informal socializing* is based only on contacts with friends and acquaintances show a slight decrease in the correlation between *informal socializing* and participation in the health check. This result is as expected, as contact with persons within both domains; family as well as friends is likely to contribute to the accumulated social capital. Lack of contact with friends may therefore be buffered from frequent family contacts [19]. Our study lacked information on cognitive aspects of social capital such as levels of interpersonal trust, perceptions of solidarity and cohesion, and willingness to help others. Second, information on individual social capital would have been relevant to include in the analysis. Again, this information was not assessable from non-participants. Including social capital at both the individual and neighborhood levels would have provided a more complete picture of the multi-level influences of social capital on our outcome.

Thanks to the access to national registers with information on all residents of the 73 neighborhoods covering the study catchment area, we were able to calculate aggregated neighborhood measures of social capital in the Inter99 study. The Danish national registers have high quality and validity [26] and such analysis is only possible in Scandinavian countries. The Inter99 study is suitable for conducting multilevel analysis with many covariates due to the large size and relatively large catchment area. Furthermore, using census districts as the neighborhood measure has several strengths. The borders of the districts are typically based on boundaries following the physical division of larger roads, division of urban and rural areas and following borders of housing associations. Because census districts are often equivalent to school districts that represent small communities distinct from one another and the relatively small size of the neighborhoods the dilution of neighborhood effects is minimized. Though the neighborhoods were sharing borders, the population density varied substantially between neighborhoods. Still, we found that including a measure of population density in the statistical model did not change the results significantly (data not presented). While most previous studies looking at effects of social capital adjust only for individual socioeconomic position, we also included neighborhood deprivation in our analysis. This illustrated that the pathway from neighborhood deprivation to participation in a health check is almost entirely mediated through social capital.

A limitation of this study is the missing data on census districts as well as missing data on educational attainment. As census areas are based on 2006 data, changes in road names during the eight year period from study start could be an explanation for the missing data on census district. In all of the 11 municipalities covering the catchment area there were persons with missing census districts, supporting this assumption. When compared to the rest of the study population, a larger proportion of the persons with missing data on education were out of work force and fewer participated in the intervention. However, there were no clear differences in regards to prevalence of ischemic heart disease, income, sex and age distribution. There exists no automatic registration on immigrants’ education level which explains most of the missing data on education. Our analyses are cross-sectional; as social capital was measured at approximately the same point in time as was participation. Voter data from the 2001 parliament election was obtained after the Inter99 health check. However, an analysis comparing the voting turnout at the election for the Danish parliament on March 11^th^ 1998 with that from 2001 showed a 0.96 correlation at the municipality level. Unfortunately, we did not have asses to voting data at the voting district level from the 1998 election; this would have assured that voting turnout was measured temporality before participation in the Inter99 health check. Nonetheless, we are confident that neighborhood characteristics at the census level are stable over time and we find it unlikely that the Inter99 study should have influenced the neighborhood voting turnout, wherefore we do not believe reverse causality to cause serious bias.

## Conclusions

In conclusion, our results suggest that the association between neighborhood social capital and participation in preventive health checks can be explained by differences in individual socioeconomic position and level of neighborhood deprivation. Nevertheless, there seems to be some residual association between social capital and health check participation, suggesting that activating social relations in the community may be an avenue for boosting participation rates in community-based health interventions.

## Additional file

Additional file 1:
**Probability (RR CI95%** ***p*** **-value) of participating by voting turnout and contact to friends and acquaintances (Model 0), plus individual factors (Model1), neighborhood deprivation (Model2) and both individual and neighborhood deprivation (model 3).**
