# Supplementary material for: Neighborhood social capital is associated with participation in health checks of a general population: a multilevel analysis of a population-based lifestyle intervention- the Inter99 study
Source: BMC Public Health. 2015 Jul 22;15:694. doi: 10.1186/s12889-015-2042-5 (PMC4511436; doi:10.1186/s12889-015-2042-5)
Supplement: Additional file 1: — Probability (RR CI95% p -value) of participating by voting turnout and contact to friends and acquaintances (Model 0), plus individual factors (Model1), neighborhood deprivation (Model2) and both individual and neighborhood deprivation (model 3). [file 12889_2015_2042_MOESM1_ESM.docx]

| **Additional file 1** Probability (RR CI95% p-value) of participating by voting turnout and contact to friends and acquaintances (Model 0), plus individual factors (Model1), neighborhood deprivation (Model2) and both individual and neighborhood deprivation (model 3) | | | | | | | | | | | | | | | | |
| --- | --- | --- | --- | --- | --- | --- | --- | --- | --- | --- | --- | --- | --- | --- | --- | --- |
|  | **Model 0** | | | | **Model 1** | | | | **Model 2** | | | | **Model 3** | | | |
|  | RR | CI95% | | P-value | RR | CI95% | | P-value | RR | CI95% | | P-value | RR | CI95% | | P-value |
| **Neighborhood level factors** | | | | | | | | | | | | | | | | |
| Informal socializing |  |  |  |  |  |  |  |  |  |  |  |  |  |  |  |  |
| *High* | 1.12 | 1.06 | 1.18 | **<0.001** | 1.06 | 1.00 | 1.12 | **0.047** | 1.08 | 1.02 | 1.15 | **0.013** | 1.04 | 0.99 | 1.11 | 0.142 |
| *Middle* | 1.08 | 1.02 | 1.14 | **0.007** | 1.04 | 0.99 | 1.10 | 0.123 | 1.07 | 1.01 | 1.13 | **0.028** | 1.04 | 0.98 | 1.10 | 0.168 |
| *Low* | 1.08 | 1.03 | 1.15 | **0.004** | 1.04 | 0.99 | 1.10 | 0.127 | 1.07 | 1.01 | 1.13 | **0.013** | 1.04 | 0.98 | 1.09 | 0.205 |
| *Very low* | 1 | (ref.) | |  | 1 | (ref.) | |  | 1 | (ref.) | |  | 1 | (ref.) | |  |
| P-value for trend | **0.002** | | | | 0.263 | | | | 0.062 | | | | 0.486 | | | |
| Voting turnout |  |  |  |  |  |  |  |  |  |  |  |  |  |  |  |  |
| *High* | 1.28 | 1.21 | 1.35 | **<0.001** | 1.13 | 1.08 | 1.20 | **<0.001** | 1.12 | 1.01 | 1.23 | **0.024** | 1.08 | 0.99 | 1.19 | 0.085 |
| *Middle* | 1.14 | 1.08 | 1.21 | **<0.001** | 1.06 | 1.00 | 1.11 | **0.043** | 1.03 | 0.95 | 1.13 | 0.463 | 1.02 | 0.94 | 1.11 | 0.608 |
| *Low* | 1.08 | 1.02 | 1.14 | **0.006** | 1.01 | 0.96 | 1.06 | 0.670 | 1.01 | 0.94 | 1.08 | 0.828 | 1.00 | 0.94 | 1.07 | 0.920 |
| *Very low* | 1 | (ref.) | |  | 1 | (ref.) | |  | 1 | (ref.) | |  | 1 | (ref.) | |  |
| P-value for trend | **<0.001** | | | | **<0.001** | | | | **0.008** | | | | 0.063 | | | |
| Neighborhood deprivation |  |  |  |  |  |  |  |  |  |  |  |  |  |  |  |  |
| *Very low* |  |  |  |  |  |  |  |  | 1.18 | 1.07 | 1.30 | **0.001** | 1.06 | 0.96 | 1.16 | 0.245 |
| *Low* |  |  |  |  |  |  |  |  | 1.13 | 1.03 | 1.23 | **0.008** | 1.04 | 0.95 | 1.13 | 0.383 |
| *Middle* |  |  |  |  |  |  |  |  | 1.07 | 1.00 | 1.15 | 0.056 | 1.00 | 0.93 | 1.07 | 0.997 |
| *High* |  |  | |  |  |  |  |  | 1 | (ref.) | |  | 1 | (ref.) | |  |
| P-value for trend |  | | | |  |  |  |  | **0.011** | | | | 0.473 | | | |
| **Individual factors** | | | | | | | | | | | | | | | | |
| Education |  |  |  |  |  |  |  |  |  |  |  |  |  |  |  |  |
| *High* |  |  |  |  | 1.17 | 1.08 | 1.27 | **<0.001** |  |  |  |  | 1.17 | 1.08 | 1.27 | **<0.001** |
| *Medium* |  |  |  |  | 1.27 | 1.20 | 1.33 | **<0.001** |  |  |  |  | 1.27 | 1.20 | 1.33 | **<0.001** |
| *Low* |  |  |  |  | 1.18 | 1.13 | 1.23 | **<0.001** |  |  |  |  | 1.18 | 1.13 | 1.23 | **<0.001** |
| *Basic* |  |  |  |  | 1 | (ref.) | |  |  |  |  |  | 1 | (ref.) | |  |
| P-value for trend |  |  |  |  | **<0.001** | | | |  |  |  |  | **<0.001** | | | |
| *Wage earner* |  |  |  |  | 1.31 | 1.26 | 1.35 | **<0.001** |  |  |  |  | 1.31 | 1.26 | 1.35 | **<0.001** |
| *Out of workforce* |  |  |  |  | 1 | (ref.) | |  |  |  |  |  | 1 | (ref.) | |  |
| P-value for trend |  |  |  |  | **<0.001** | | | |  |  |  |  | **<0.001** | | | |
| Income |  |  |  |  |  |  |  |  |  |  |  |  |  |  |  |  |
| *I-Highest quartile* |  |  |  |  | 1.32 | 1.25 | 1.40 | **<0.001** |  |  |  |  | 1.32 | 1.25 | 1.40 | **<0.001** |
| *II* |  |  |  |  | 1.22 | 1.15 | 1.29 | **<0.001** |  |  |  |  | 1.21 | 1.15 | 1.28 | **<0.001** |
| *III* |  |  |  |  | 1.16 | 1.10 | 1.23 | **<0.001** |  |  |  |  | 1.16 | 1.10 | 1.23 | **<0.001** |
| *IV-lowest quartile* |  |  |  |  | 1 | (ref.) | |  |  |  |  |  | 1 | (ref.) | |  |
| P-value for trend |  |  |  |  | **<0.001** | | | |  |  |  |  | **<0.001** | | | |
